# Vine-Canes Valorisation: Ultrasound-Assisted Extraction from Lab to Pilot Scale

**DOI:** 10.3390/molecules25071739

**Published:** 2020-04-10

**Authors:** Olena Dorosh, Manuela M. Moreira, Francisca Rodrigues, Andreia F. Peixoto, Cristina Freire, Simone Morais, Cristina Delerue-Matos

**Affiliations:** 1 REQUIMTE/LAQV, Instituto Superior de Engenharia do Instituto Politécnico do Porto, Rua Dr. António Bernardino de Almeida, 431, 4249-015 Porto, Portugal; o.dorosh@campus.fct.unl.pt (O.D.); franciscapintolisboa@gmail.com (F.R.); sbm@isep.ipp.pt (S.M.); cmm@isep.ipp.pt (C.D.-M.); 2 REQUIMTE/LAQV, Departamento. de Química e Bioquímica, Faculdade de Ciências, Universidade do Porto, Rua do Campo Alegre s/n, 4169-007 Porto, Portugal; andreia.peixoto@fc.up.pt (A.F.P.); acfreire@fc.up.pt (C.F.)

**Keywords:** vine-canes, ultrasound-assisted extraction, pilot scale, polyphenols, antioxidant activity, HPLC-PDA analysis

## Abstract

Wine production generates large amounts of vine-canes, a devalued by-product that could be used for the recovery of bioactive compounds. In this work, two vine-canes varieties, namely Touriga Nacional (TN) and Tinta Roriz (TR), were submitted to different ultrasound-assisted extraction (UAE) conditions. The highest phenolic and flavonoid content was observed for TR extract obtained at lab-scale without an ice bath and pilot-scale after 60 min of extraction (32.6 ± 2.1 and 26.0 ± 1.5 mg gallic acid equivalent/g dry weight (dw) and 9.5 ± 0.6 and 8.3 ± 0.8 mg epicatechin equivalents/g dw, respectively). Further, all extracts demonstrated a high antioxidant activity to scavenge DPPH free radicals with the best value reached by TR at the lab-scale without an ice bath after 30 min and pilot-scale extraction after 60 min (34.2 ± 2.4 and 33.4 ± 2.1 mg trolox equivalents/g dw, respectively). Extracts phenolic composition were also evaluated by HPLC, demonstrating that resveratrol, myricetin and catechin were the main compounds. According to our knowledge, this is the first time that a pilot scale of UAE of phenolic compounds from vine-canes was performed. This paper represents an important step to the use of UAE as an industrial process to recover bioactive compounds.

## 1. Introduction

Grapes are one of the major fruit crops produced throughout the world [1], and in 2018 approximately 57% of the harvested grapes were used to make wine [2]. According to the International Organization of Vine and Wine (OIV), Portugal is the country with the 11th highest wine production, being viticulture one of Portuguese’s major economic sectors [2]. It is estimated that one hectare of vineyard leads to the production of approximately 1.75 tons of vine-cane pruning, depending on the vine varieties [3]. In 2016, the amount of vine-canes produced corresponded to approximately 3.4 × 10^5^, 6.4 × 10^6^ and 1.3 × 10^7^ tons in Portugal, Europe and globally, respectively [3,4]. Usually, after the harvesting season, vine-canes are burned [5] or incorporated in the soil, which due to the degradation of organic matter, enhances the soil health reducing the need to apply organic fertilizers and/or correctives [6]. Even so, there has been a search for more profitable applications for vine-canes, and several alternatives were already investigated, such as the production of biochars, biofuels, pulp for paper sheets, particle board and lignin [7,8,9,10,11]. Recently, several studies have demonstrated that vine-canes are rich in polyphenols [12,13,14,15,16,17] and could be used as a source of these bioactive compounds.

The main phenolic compounds present in vine-canes belong to six different classes: (1) flavanols (catechin and epicatechin); (2) phenolic acids (gallic, *trans*-caftaric, *trans*-*p*-coutaric, ellagic, syringic, chlorogenic and vanillic acids); (3) stilbenes (resveratrol); (4) flavanones (naringin, naringenin and pinocembrin); (5) flavonols (rutin, myricetin, kaempeferol and quercetin) and (6) condensed tannins (proanthocyanidins) [12,14,17,18,19]. Due to the high variability in plant materials and phenolic structures, the profile and the yield of the recovered phenolic compounds can be affected by the extraction technique and by experimental conditions, such as temperature, time, solvent composition, solid-liquid ratio and particle size [15]. Currently, conventional extraction (CE) methods, such as hydro-distillation, Soxhlet, steam distillation, maceration and infusion, are the most employed processes to recover phenolic compounds from natural products [20]. However, they present some drawbacks, such as a low extraction efficiency, time consuming, use of large amounts of solvents that generally are not environmentally friendly and require the employment of high temperatures (which in certain cases could lead to the degradation of some thermolabile compounds such as anthocyanins), which all together also results in a high energy consumption [12,21]. Therefore, emerging environmentally friendly technologies, including ultrasound-assisted extraction (UAE), have been proposed as more sustainable and efficient methods for the recovery of bioactive compounds [12,21,22].

UAE can be performed using devices that transmit ultrasound radiation, such as the simple water baths, probes, sonoreactors, and microplate horns [23]. The energy produced by the sound waves propagates into the liquid media (with frequencies around 20 kHz) creating two cycles that occur alternatively: (1) a cycle of high pressure (called compression) and (2) a low pressure cycle (called rarefaction); series of these cycles create an acoustic pressure [15]. During the rarefaction, tiny vapor bubbles (voids) are produced, that grow during the cycles until they can no longer absorb energy, suffering an implosive collapse called cavitation [24]. In plant samples, the implosive collapse leads to an enlargement in the pore walls or the disruption of the cell walls in a short period of time, promoting the penetration of the solvent into the sample and releasing the target compounds. As a result, the mass transfer across cell membranes is enhanced, increasing the extraction efficiency [25]. This technique reduces the time needed for the extraction, the solvent consumption by improving the surface contact area between the matrix and the extraction solvent, while it also increases the yield [26].

To the best of our knowledge, only two papers reporting the application of UAE, using a probe, for the extraction of phenolic compounds from vine-canes were found [5,14]. Delgado-Torre et al. [14] performed UAE in 18 different vineyard cultivars from Sierra de Segura (Spain) and compared the extraction efficiency with a superheated liquid extraction (SHLE) and microwave-assisted extraction (MAE). These authors concluded that SHLE enables to recover the highest quantity of phenolic compounds in comparison to UAE and MAE techniques (650.4, 546.4 and 401.4 µg gallic acid equivalents (GAE)/mL extract, respectively). Piñeiro et al. [5] optimized the pre-treatment and UAE-based method for the extraction of stilbenes from 20 vine-canes varieties. A 10 min extraction, 1:40 (*w*/*v*) sample-solvent ratio, 60% (*v*/*v*) aqueous ethanol at 75 °C allowed to obtain 3558.7 ± 81.4 mg of total stilbenes per kg of dry weight (DW). Other studies also employed ultrasound to enhance the extraction of bioactive compounds. Nevertheless, these studies either used ultrasound as a pre-treatment, and not in the extraction itself; or in sonication baths, which are not as reproducible as ultrasound probes [15,27,28].

The present work is focused on evaluating the efficiency of extracting phenolic compounds by UAE from two Portuguese vine-canes varieties, namely Touriga Nacional (TN) and Tinta Roriz (TR) from the Dão region. UAE was performed at a laboratory scale and the influence of the use of an ice bath in the extraction efficiency was also evaluated. Moreover, for the first time, a scale up of the UAE process was also performed enabling to evaluate the potential of this extraction technique for industrial applications. All the obtained extracts were characterized spectrophotometrically, through total phenolic content (TPC) and total flavonoid content (TFC), as well as antioxidant activities assays, namely by 2,2-diphenyl-picrylhydrazyl radical scavenging activity (DPPH-RSA) and Ferric Reducing Antioxidant Power (FRAP) assays. The individual phenolic compounds contributing to the composition of the obtained extracts were also determined by high performance liquid chromatography with photodiode array detection (HPLC-PDA).

## 2. Results and Discussion

### 2.1. Total Phenolic and Flavonoid Content and Antioxidant Activity

Table 1 summarizes the TPC, TFC and antioxidant activity of the obtained extracts. For each result, the influence of different parameters, such as the *V. vinifera* vine-cane variety, the extraction time, the use or not of an ice bath at lab scale experiments and the scaling up of the process, were evaluated.

#### 2.1.1. Total Phenolic and Flavonoid Content

According to Table 1, for all the extraction conditions tested, TR variety presented higher TPC and TFC than TN variety (*p* < 0.05), except for UAE performed at lab scale with an ice bath (16.2 ± 2.1 and 17.1 ± 1.3 mg GAE/g DW for TN and TR varieties, respectively). The highest amount of phenolic and flavonoid compounds was quantified for TR extracts from UAE performed at lab scale without an ice bath after 60 min of extraction (32.6 ± 2.1 mg GAE/g DW and 9.5 ± 0.6 mg EE/g DW, respectively), which was 1.6 and 1.8 fold higher than the value obtained for TN variety. Several factors may affect the phenolic profile of grapes, as the geographical location, the climate, the presence of abiotic stress and the grape variety [29,30]. Sen et al. [29] characterized and compared the wines of 11 grape varieties and concluded that the variety exerts more influence in the phenolic profile of wines than the geographical location. The results obtained for the UAE are in accordance with the conclusions reported by Sen et al. [29], demonstrating that the two Portuguese vine-canes varieties grown under the same conditions presented considerable differences in the TPC and TFC values, indicating that these differences may be due to the vine-cane variety.

Regarding the influence of extraction time, significant differences (*p* < 0.05) were also observed for the majority of extraction times tested, for both vine-cane varieties. In fact, when a comparison for the highest values is performed, namely for UAE at lab-scale without an ice bath, an increase of 9.3 times was observed for the extractions carried out during 60 min vs. those made for 30 min, while an increase of only 4.6 times was recorded when the extraction time increased from 15 to 30 min. Although, through the analysis of Table 1, it is possible to observe in generally a higher difference between the phenolic and flavonoid content for the extractions performed for 15 and 30 min, than between 30 and 60 min. These results demonstrate that longer extraction times did not improve the yield of phenolic compounds, suggesting that 30 min could be enough for the extraction of the majority of phenolic compounds, avoiding an higher consumption of energy and time which reflects in money saving [19,22,25].

The temperature also appears to affect the amount of extracted phenolic compounds (Table 1). The UAE performed at lab scale without an ice bath (62 to 76 °C) or at pilot scale (64 to 70 °C) enabled to obtain higher values of total phenolic and flavonoid content than the extraction performed at lab scale with an ice bath (32 to 46 °C), this leads us to conclude that higher extraction temperatures seem to have a positive effect on the recovery of phenolics from vine-canes. Higher temperatures could weaken the linkage between polyphenols and polysaccharides, releasing phenolic compounds of low molecular weight and increasing the solubility of solutes and the diffusion coefficient [12,31]. Sánchez-Gómez et al. [12] compared the extraction efficiency of phenolic compounds from vine-canes by conventional solid-liquid extraction (CSLE; at ca. 100 °C) and solid-liquid dynamic extraction–Naviglio (SLDE-Naviglio; at 25–27 °C). These authors reported a 4-fold and 20-fold increase in flavonols and *trans*-resveratrol content using the CSLE, which was probably caused by the higher temperature used in this method in comparison to the SLDE-Naviglio. 

In another study, Karacabey et al. [31] also reported that using lower or moderate ethanol concentrations (50–70%) the increase in the extraction of phenolic compounds from vine-canes was practically linear with the increase of temperature. These conclusions are in agreement with the results obtained in the present study, since the highest phenolic and flavonoid contents were reported for the UAE performed at the highest temperatures (lab scale without an ice bath and pilot scale).

Comparing the efficiency of UAE performed at lab scale with the extraction performed at pilot scale, the highest TPC and TFC, independently of the time and the vine-cane variety considered, was obtained at lab scale without an ice bath (32.6 ± 2.1 mg GAE/g DW for TR variety). Nevertheless, the obtained results suggest that a scale-up of the UAE might not have a major impact on the amount of the total phenolic and flavonoid compounds recovered from vine-canes. In fact, UAE performed at pilot scale seems to be more profitable when employed to TR vine-cane variety, since the TPC and TFC were similar to the ones obtained for lab scale without an ice bath (corresponding usually to the highest values). This is the first study reporting the comparison between the UAE performed at lab scale with an ultrasound probe *versus* the UAE performed at larger scale, and the reported results are extremely important from an industrial point of view, as they demonstrated that the proposed extraction technique could be used at industrial scale without compromising the polyphenols recovery efficiency.

As previously mentioned, only two studies reporting the use of UAE probes for the recovery of phenolic compounds from vine-canes were found, being one of them exclusively focused on the stilbenes compounds [13,26]. Delgado-Torre et al. [14] compared the efficiency of three environmentally friendly extraction techniques, namely SHLE, MAE and UAE, reporting that SHLE allowed to obtain the highest TPC (650.4 µg/mL). Nevertheless, good results were also obtained for the UAE (546.4 µg/mL), while MAE appeared to be the less effective (401.4 µg/mL). Farhadi et al. [28] also performed UAE (but using an ultrasonic bath) of skins, pulp, seeds, canes and leaves from one international and five native grape varieties cultivated in west Azerbaijan (Iran). Vine-canes extracts provided the best results after grape skins, and leaf extracts exhibited the lowest TPC. In the case of vine-cane extracts, the highest TPC was approximately 250 mg GAE/g DW, which is almost 7.7-fold higher than the value obtained in the present work. Nevertheless, these authors used a less environmentally friendly solvent (methanol/HCl 99/1, *v*/*v*). The same authors [28] also determined the TFC of the extracts, unfortunately a direct comparison cannot be established as the authors used quercetin as a standard. Nevertheless, from the different matrices analyzed by Farhadi et al. [28], vine-canes were the best source to recover flavonoid compounds. Additionally, a direct comparison of the results from the present study with the ones from our previous work can also be performed [17]. Previously, the highest TPC was also obtained for TR variety employing MAE, followed by subcritical water extraction (SWE) and CE (32.1 ± 0.9, 31.9 ± 1.6 and 25.9 ± 0.9 mg GAE/g DW, respectively). The TPC obtained in the present study by UAE performed at lab scale without an ice bath (32.6 ± 2.1 mg GAE/g DW) was very similar to the values reported before [17]. Although, as one of the main goals of the present work was to demonstrate the feasibility of a scaling up of the extraction process, and as MAE is a extraction technique difficult to scale-up, in the future it could be very interesting to compare the efficiency of UAE at pilot scale to a scale up of SWE in order to select the most appropriate technique to be explored at an industrial scale for the recovery of phenolic compounds from vine-canes. Regarding the TFC, Moreira et al. [17] reported a two-fold higher content for TR vine-cane variety using the SWE than the highest value obtained by UAE, indicating that if the goal of the work is to obtain compounds belonging to flavonoid family, SWE can be a better choice than UAE.

#### 2.1.2. DPPH-Radical Scavenging Activity Assay

The antioxidant activity of the obtained extracts was evaluated by the DPPH-RSA assay. In accordance with the obtained results for phenolic and flavonoid content (Table 1), TR vine-canes variety also presented the highest antioxidant activity (*p* < 0.05), independently of the used UAE conditions. In contradiction to the conclusions obtained through the analysis of TPC and TFC results, it seems that high temperatures result in a negative effect on the antioxidant capacity of the extracts. As it can be seen (Table 1), the extracts obtained at lab scale with an ice bath kept increasing their antioxidant activity over the extraction time; however, the antioxidant activity without an ice bath or at pilot scale was lower for extraction times longer than 30 min. These results could be related with the degradation of some compounds caused by the increase of temperature during the UAE [12], as it was already discussed in the Section 2.1.1. Regarding the influence of the scaling up in the antioxidant activity of the extracts, it can be also concluded that the scale up of the UAE for an extraction time up to 30 min could be more profitable for TN vine-cane variety. On the other hand, for TR vine-cane variety the scale up (from the lab scale without an ice bath to a pilot scale) negatively affected the antioxidant activity (*p* < 0.05). A possible explanation for these differences between the vine-cane varieties could be related with the phenolic composition of each variety, which will be explored and discussed in Section 2.2.

The antioxidant activity of vine-canes has been previously investigated by several authors [14,16,22,27,28,32]. Unfortunately, most of those studies used different assays to evaluate the antioxidant activity and even when the DPPH method was used the results were usually expressed in terms of inhibition percentage (IC_50_). Consequently, it is only possible to directly compare the values obtained by UAE with the results previously reported by Moreira et al. [17]. According to these authors [17], SWE enables to obtain vine-cane extracts with the highest antioxidant activity (24.8 ± 1.9 and 35.5 ± 2.4 mg TE/g DW for TN and TR varieties, respectively), which were more efficient on scavenging DPPH radicals than the extracts obtained by UAE. Still, in some cases for TR variety, no significant differences were observed between the results reported by Moreira et al. [17] and the extracts obtained by UAE performed at lab scale without an ice bath or at pilot scale (34.2 ± 2.4 and 33.4 ± 2.1 mg TE/g DW, respectively).

#### 2.1.3. Ferric Reducing Antioxidant Power assay

The results obtained for the FRAP method agreed with the ones obtained for the TPC, TFC and DPPH-RSA assays (Table 1). TR extracts presented higher reducing power than TN vine-cane variety (*p* < 0.05), except for the UAE performed at lab scale with an ice bath for 60 min where no statistically significant differences (*p* > 0.05) between TN and TR were observed. According to the obtained results, longer extraction periods positively affected the extracts capacity to reduce Fe^3+^ to Fe^2+^, with the highest FRAP values being obtained for UAE performed for 60 min. Regarding the influence of the scaling up of the UAE on reducing power of vine-cane extracts, no significant differences were obtained for FRAP values, except for the highest extraction time employed (60 min). As aforementioned, the reported differences in FRAP values can be related with the extract’s composition, which can be affected not only by phenolic compounds but also due to the formation of new compounds that can be contributing to the antioxidant properties.

As previously mentioned for DPPH-RSA assay, no reports were found that enable direct comparison of the present values with the ones from the literature. Although, a comparison can be made with the results from Moreira et al. [17]. The FRAP values obtained by UAE were lower than the ones reported for SWE (20.2 ± 1.7 and 24.3 ± 0.9 mg AAE/g DW for TN and TR, respectively) by these authors [17]. Taking into consideration the above-mentioned results, in the future, it could be very interesting to perform a scale up of the SWE and then compare the obtained results with the ones from UAE performed at pilot scale. These data could be very useful to demonstrate to winery industries the potential value of vine-canes pruning by recovering bioactive compounds. Although, an evaluation of the economic and environmental impact from the scale-up process is also needed to evaluate the applicability of the proposed extraction technique at an industrial scale.

### 2.2. Identification of Individual Phenolic Compounds by HPLC-PDA

**Figure 1:**
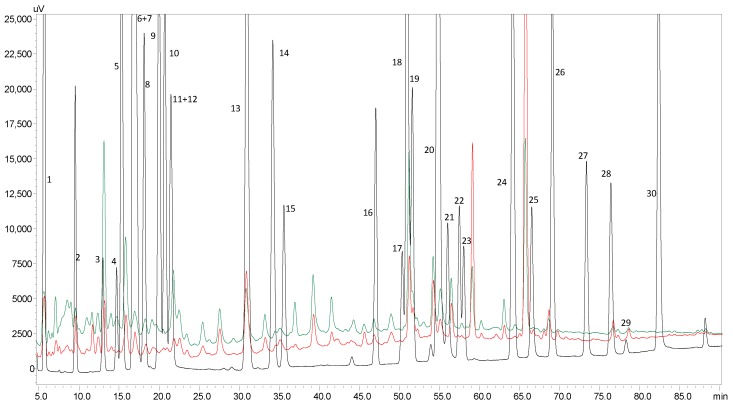
HPLC chromatograms at 280 nm for: polyphenols standard mixture of 10 mg/L (black line), and ultrasound-assisted extracts obtained at lab scale without an ice bath after 60 min of extraction for TN (Touriga Nacional, red line) and TR (Tinta Roriz, green line) vine-cane varieties; (1) gallic acid, (2) protocatechuic acid, (3) (+)-catechin, (4) 4-hydroxyphenilacetic acid, (5) 4-hydroxybenzoic acid, (6) 4-hydroxybenzaldehyde, (7) chlorogenic acid, (8) vanillic acid, (9) caffeic acid, (10) syringic acid, (11) (−)-epicatechin, (12) β-resorcylic acid, (13) *p*-coumaric acid, (14) ferulic acid, (15) sinapic acid, (16) naringin, (17) rutin, (18) resveratrol, (19) quercetin-3-*O*-glucopyranoside, (20) phloridzin, (21) cinnamic acid, (22) myricetin, (23) kaempferol-3-*O*-glucoside, (24) kaempferol-3-*O*-rutinoside, (25) naringenin, (26) quercetin, (27) phloretin, (28) tiliroside, (29) kaempferol and (30) pinocenbrim.

In order to identify and quantify the individual phenolic compounds of vine-canes, and to understand which compounds could be correlated with the antioxidant properties of the obtained extracts, HPLC analyses were performed. Taking into consideration the previous results obtained by the spectrophotometric assays, the extracts analyzed by HPLC-PDA were the ones obtained by UAE at lab scale without an ice bath for 60 min. Figure 1 exhibits representative UV chromatograms at 280 nm and Table 2 displays the individual phenolic compounds content for each vine-cane variety.

As it can be observed from the results presented in Table 2, the highest total amount of phenolic compounds was quantified for TR vine-cane variety, which was already expected considering the results previously obtained for the spectrophotometric assays. Phenolic acids were present in similar amounts for both vine-canes varieties, which is in accordance with the results reported by Moreira et al. [17]. In another study [28], only gallic acid was identified and quantified in vine-cane extracts obtained by an ultrasonic bath (content ranged between 10.2 ± 0.4 and 15.3 ± 0.8 mg of gallic acid/100 g DW), which was similar to the values reported in the present study for TN and TR varieties (Table 2).

Regarding the identification and quantification of flavanols, 3.5-fold higher amounts were found in TR variety than in TN (Table 2). For both vine-cane varieties, catechin was quantified in higher amounts than epicatechin. This difference was also observed by Farhadi et al. [28] concerning the skins, pulp and canes of vines, but it was not very significant in the case of seeds and leaves. The previous authors were able to extract 51.3 ± 2.8 mg of catechin/100 g DW and 23.4 ± 1.6 mg of epicatechin/100 g DW from vine-canes using an ultrasound bath, which were 2.6-fold lower and 1.3-fold higher than the values obtained for TR in the present study. The differences in the quantified flavanols is probably caused by the solvent composition, which was already proved to be one of the parameters that affects the quality and quantity of the phenolic compounds recovered [15]. Moreira et al. [17] also reported similar amounts of flavanols for the CE and MAE extracts as the ones obtained in the present study for UAE. The common factor in the three referred techniques is the solvent composition, which was very similar (ethanol/water 50/50 for CE and UAE, and 60/40 for MAE) supporting the idea that the solvent composition highly affects the extraction of this subclass of phenolic compounds.

The compounds belonging to the flavonols family were also huge contributors for the phenolic composition in both vine-cane varieties, representing approximately 42% of the total amount of phenolic compounds quantified in TN and 29% in TR variety. Myricetin (74.9 ± 3.7 and 74.3 ± 3.7 mg of myricetin/100 g DW, for TN and TR respectively) was the flavonoid compound present in higher amount, followed by kaempferol (23.9 ± 1.2 and 22.8 ± 1.1 mg of kaempferol/100 g DW, for TN and TR respectively.), rutin (23.3 ± 1.2 and 21.8 ± 1.1 mg of rutin/100 g DW, for TN and TR respectively) and quercetin (20.1 ± 1.0 and 19.7 ± 1.0 mg of quercetin/100 g DW, for TN and TR respectively) in very similar quantities. These compounds were already identified by other authors in vine-canes, leaves, wine lees, pulp as well as in grape skins and seeds [17,28,33,34]. Farhadi et al. [28] also quantified the amount of some flavonols in vine-canes reporting 3.0 ± 0.3 mg of rutin/100 g DW and 95.6 ± 8.7 mg of quercetin/100 g DW, which were 7.8-fold lower and 4.8-fold higher than the results obtained in this study. Despite of the previous authors used an ultrasonic bath instead of a probe, the main difference in the referred values may be explained not only by the used of different vine-cane varieties but also due to the solvent composition employed in each study. On the other hand, by comparing our data to the values reported by Moreira et al. [17], which used the same vine-canes varieties, higher amounts of flavonols compounds were extracted by these authors employing MAE (712 ± 35 and 680 ± 34 mg of flavonols/100 g DW for TN and TR, respectively) than the content obtained in the present study by UAE (Table 2). The difference between the two studies is the employed extraction technique, which in the case of Moreira et al. [17] appears to be more efficient to recover higher amounts of these type of compounds.

Resveratrol is a wine polyphenol commonly associated to the prevention of cardiovascular diseases and also to increase stress resistance and lifespans [35]. This compound was one of the major contributors to the phenolic profile for both vine-canes varieties, being the content reported for TR variety 1.8-fold higher than in TN. The amount of resveratrol obtained through UAE was higher than the values reported by Moreira et al. [17] for any of the extraction techniques tested. The values reported by these authors were 8.9-fold and 16.3-fold lower than the ones obtained for TN and TR by UAE technique, respectively. Furthermore, Frahadi et al. [28] also reported low quantities of resveratrol for all the vine-cane varieties tested (the best value was 0.26 mg/100 g DW), suggesting that the use of acidic methanol as extracting solvent was not indicated for the recovery of this stilbene compound. In fact, the results previously discussed enable to conclude that not only the solvent composition but also the extraction technique employed seems to exert a huge influence on the amount of flavonoid compounds recovered.

It is well described in the literature [36] that phenolic compounds, specially belonging to the flavonoid family, possess high antioxidant and anti-inflammatory activity, as well as the capacity to reduce the risk in developing diseases related with chronic inflammation, namely cancer, neurodegenerative and cardiovascular diseases [37,38]. Therefore, the potential application of vine-canes in the food, cosmetic or pharmaceutical industries could represent an added value for the winery companies. Moreover, the results of this investigation propose UAE process as a potential way for scaling up either ultrasound [39] or hydrodynamic cavitation [40] allowing a process intensification.

## 3. Materials and Methods 

### 3.1. Chemicals

Absolute ethanol (≥99%) used in the extractions performed with ultrasound was obtained from Fisher Chemical (Loughborough, UK). 2,2-Diphenyl-1-picrylhydrazyl radical (DPPH˙), (±)-6-hydroxyl-2,5,7,8-tetramethylchromane-2-carboxylic acid (Trolox^®^), Folin’s and Ciocalteu’s (FC) phenol reagent and sodium carbonate (≥99%), used in the spectrophotometric assays were purchased from Sigma-Aldrich (Madrid, Spain). 2,4,6-Tris(2-pyridyl)-s-triazine (TPTZ, 99%), (−)-epicatechin (≥90%), gallic acid monohydrate (GA, ≥98%) and hydrochloric acid (≥37%) were obtained from Fluka (Munich, Germany). Aluminium chloride (99.52%) and sodium acetate 3-hydrate (99%) were acquired at Panreac (Barcelona, Spain). Iron(III)chloride-6-hydrate (≥99%) and L(+)- ascorbic acid (≥99.7%) were from Riedel-de Haën (Seelze, German), glacial acetic acid (≥99.5%) was from Carlo Erba (Peypin, France), sodium nitrite (98%) from M&B Chemicals (City Road, London) and sodium hydroxide (>99%) from Labkem (Zelienople, Pennsylvania). For HPLC analysis, methanol (≥99.8%) and formic acid (≥98%) gradient grade were from VWR (Lisboa, Portugal), BDH (Dubai, UAE), Probalo (Fontenay Sous Bois Cedex, France) and Merck (Darmstadt, Germany), respectively. Individual phenolic compounds standards were purchased from Sigma-Aldrich and their purity was at least above 95%.

### 3.2. Vine-Cane Samples

The *Vitis Vinifera* vine-canes varieties, gently provided by Sogrape Vinhos, S. A. (Porto, Portugal), employed in the present work were TN and TR, whose references are preserved in the living reference collection “Colecção Ampelográfica Nacional”, managed by the National Institute for Agricultural and Veterinary Research, in “Dois Portos”, with the numbers: PRT52206 for TN and PRT52603 for TR (or Aragonez or Tempranillo). Samples were randomly collected at Quinta dos Carvalhais, located in Mangualde (North of Portugal) in November of 2015, after grape harvesting and leaf fall. Both varieties have been cultivated under the same environmental hydric conditions. The vine-canes were then oven-dried (Model no. 2000208, J.P. Selecta, Barcelona, Spain) at 50 °C for 24 h, and milled (ZM200, Retsch, Porto, Portugal) to a particle size smaller than 1 mm. The milled vine-canes were stored in polyethylene sealed bags at room temperature until use.

### 3.3. Ultrasound-Assisted Extraction: Laboratorial and Pilot Scale 

In the present study, the experimental domain was defined taking into account the results obtained in previous studies [5,15,38], as well as the operative limits of the equipment’s used. The sample:solvent ratio was selected considering the experimental results from Piñeiro et al. [5], while the extraction solvent was chosen considering the results obtained by other authors [15,38].

**Figure 2:**
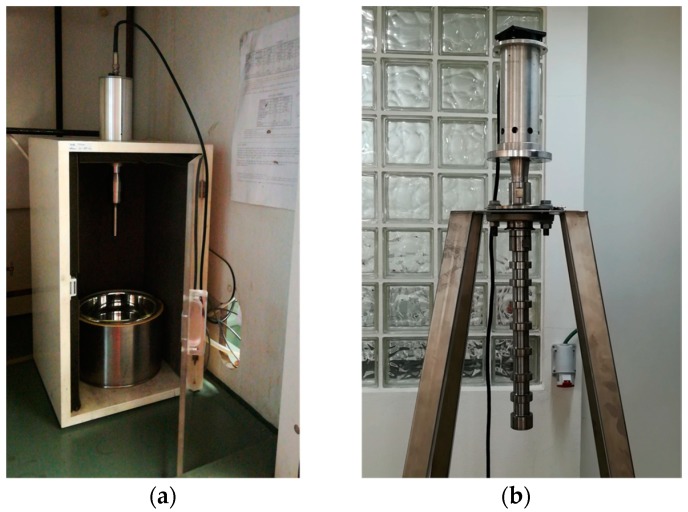
Probes used for the ultrasound-assisted extraction at: (**a**) lab scale; (**b**) pilot scale.

UAE at laboratory scale (Figure 2a) was performed with a VCX500 ultrasonic processor (Vibra-Cell, Newton, Massachusetts, USA, 500 W and 20 kHz) connected to a probe tip NO. 630-0220 with 13 mm diameter. For these extractions 4.0 g of milled vine-canes and 200 mL of ethanol:water 50:50 (*v*/*v*) were placed in a glass beaker, and the procedure was executed with and without an ice bath [5,15,38]. The use of ice aimed to minimize the temperature increase resulting from the propagation of ultrasound waves.

The pilot scale (Figure 2b) UAE was performed with an IncBio ultrasonic processor 3 kW and 20 kHz) connected to a probe made from titanium with coating material and component protection from aluminum (55 mm diameter). UAE was carried out by placing 30 g of milled vine-canes with 1500 mL of ethanol:water 50:50 (*v*/*v*). These extractions were conducted at the IncBio company located in Maia (Portugal) [41], which kindly provided all the equipment necessary to perform them.

UAE performed at lab and pilot scale were carry out for 60 min with a frequency of 20 kHz and every fifteen minutes an aliquot of 1.5 mL was collected, and the extraction temperature was recorded. The UAE conditions were tested in triplicate for each vine-cane variety. The obtained extracts were centrifuged at 15,763× *g* (Heraeus Megafuge 16 Centrifuge Series, Thermo Scientific, Massachusetts, USA) for 15 min at 4 °C. After centrifugation, the supernatant was stored in the freezer at −20 °C until further analysis. The scale-up criterion adopted in this study was to maintain constant the ratio between solid vine-canes and extracting solvent (1:50), considering that no differences will be caused in the mechanism of this process.

### 3.4. Determination of Total Phenolic and Flavonoid Content and Antioxidant Activity

To evaluate the extraction efficiency, the TPC, TFC, DPPH-RSA and FRAP assays were performed for all the obtained extracts. Absorbance measurements were made on a Synergy HT microplate reader (BioTek Instruments, USA) using the Gen5 2.00 program, and all the analyses were performed in triplicate.

#### 3.4.1. Total Phenolic and Flavonoid Content

TPC assay, based on the Singleton and Rossi original method [42], was performed as described by Paz et al. [43]. In this assay, the Folin–Ciocalteau’s reagent composed of tungsten and molybdenum changes its color from yellow to blue after reacting with reducing species, under alkaline conditions [36]. Results were expressed as mg gallic acid equivalents (GAE) per g of DW of milled vine-canes (mg GAE/g DW). TFC method measures the formation of the flavonoid-aluminium compound [44]. The assay was performed according to Paz et al. [43]. Results were expressed as mg epicatechin equivalents (EE) per g of DW of milled vine-canes (mg EE/g DW).

#### 3.4.2. DPPH-Radical Scavenging Activity Assay

DPPH-RSA was performed following the protocol described by Paz et al. [43] using Trolox^®^ to perform the calibration curve. Results were expressed in mg of Trolox^®^ equivalents (TE) per g of DW of milled vine-canes (mg TE/g DW).

#### 3.4.3. Ferric Reducing Antioxidant Power Assay

The original FRAP assay developed by Benzie and Strain [45] was performed with some modifications as described by Paz et al. [43]. Results were expressed as mg of ascorbic acid equivalents (AAE) per g of DW of milled vine-canes (mg AAE/g DW).

### 3.5. High Performance Liquid Chromatography Analysis 

The characterization and quantification of polyphenols in the vine-cane extracts were carried out by HPLC-PDA. HPLC analyses were performed using the method previously described and validated by Moreira et al. [46] on a Shimadzu HPLC system (Shimadzu Corporation, Kyoto, Japan) equipped with a Gemini C_18_ column (250 mm × 4.6 mm, 5 μm, Phenomenex, Alcobendas, Spain) and a guard column with the same characteristics at 25 °C. Individual stock solutions of the standards were prepared in methanol at concentration levels of 2000 mg/L and stored at −20 °C. For calibration curves (1 to 200 mg/L), the stock standards were dissolved in appropriate amounts in a mixture of methanol:water 50:50 (*v*/*v*). The mobile phase was composed by methanol (A) and water (B) both with 0.1% formic acid at a flow rate of 1.0 mL/min. The amount of individual phenolic compounds found in each extract, resulting from duplicate injections, was expressed as mg of compound/100 g of DW.

### 3.6. Statistical Analysis

SPSS 24.0 software (SPSS Inc., Chicago, IL, USA) was used for the statistical analysis of the obtained results from the TPC, TFC, DPPH-RSA and FRAP assays, where the data was reported as mean ± standard deviation (SD) of three replications. The normal distribution and the homogeneity of variances were assessed by Shapiro-Wilk’s and Levene’s tests, respectively. For all assays, the data were normally distributed. To evaluate the differences between samples, the one-way ANOVA was used. Tukey’s HSD test was employed for the post hoc comparisons of the means. To compare the same sample at different temperatures, a t-test was employed. Statistical significance was set at *p* < 0.05 level.

## 4. Conclusions

This work focused on employing a sustainable extraction technique for the valorization of Portuguese vine-canes. However, and considering the importance of this agro-industrial by-product derived from wine industry at the world level, it is expected that the same extraction technique could be employed in other vine-cane varieties from other geographic regions with the same success. Various parameters, such as extraction time and temperature, critical in the phenolic compounds UAE were investigated. For all the extracts analyzed, higher extraction times and temperatures resulted in higher total phenolic and flavonoid content as well as higher antioxidant activity, with TR variety reaching a maximum TPC of 32.6 ± 2.1 mg GAE/g DW (60 min of lab scale extraction). When compared to the extractions performed at lab scale without an ice bath, the scale up showed to be an interesting improvement of the UAE to be performed in industries, since the amount of phenolic compounds did not decrease substantially and in addition allowed to obtain 7.5-fold higher quantities of extract using the same extraction time. Despite of the use of LC-MS/MS could be precious to undoubtedly identify the target compounds in vine-cane extracts, HPLC analysis enable to conclude that they were mainly composed by resveratrol, catechin and myricetin; in fact, these compounds correspond to 44 and 60% of the total amount of individual phenolic compounds quantified in TN and TR varieties, respectively. This exploitation of vine-canes could present a significant environmental impact in the sustainability of food and beverage industries, since the same crop plantations could be simultaneously used to produce food and to obtain bioactive compounds with less resources including arable land and water.

## Figures and Tables

**Table 1:** Total phenolic content (TPC, mg gallic acid equivalents/g dry weight), total flavonoid content (TFC, mg epicatechin equivalents/g dry weight), 2,2-diphenyl-1-picrylhydrazyl radical scavenging activity (DPPH-RSA, mg trolox equivalents/g dry weight) and ferric reduction activity power (FRAP, mg ascorbic acid equivalents/g dry weight) of ultrasound-assisted extracts from Touriga Nacional (TN) and Tinta Roriz (TR) vine-canes varieties from Dão Region. Results are expressed as mean ± standard deviation (*n* = 3).

Time (min.)	UAE Technique		TPC (mg GAE/g DW)		TFC (mg EE/g DW)		DPPH-RSA (mg TE/g DW)		FRAP (mg AAE/g DW)	
			TN	TR	TN	TR	TN	TR	TN	TR
15	Lab scale	With ice bath	9.3 ± 0.3 ^c,3^	14.9 ± 0.7 ^b,3,^*	2.32 ± 0.19 ^c,3^	4.5 ± 0.26 ^c,2,^*	6.1 ± 0.3 ^c,2^	15.7 ± 0.7 ^c,3,^*	5.2 ± 0.5 ^c^	10.2 ± 0.6 ^2,^*
		Without ice bath	15.5 ± 0.5 ^c,1^	18.7 ± 1.2 ^c,2,^*	3.78 ± 0.44 ^b,1^	6.9 ± 0.6 ^c,1,^*	12.5 ± 1.4 ^c,1^	29.2 ± 1.6 ^b,1,^*	5.2 ± 0.4 ^c^	12.4 ± 0.9 ^b,1,^*
	Pilot scale		10.6 ± 1.2 ^b,2^	20.6 ± 1.1 ^b,1,^*	2.87 ± 0.25 ^b,2^	6.5 ± 0.5 ^c,1,^*	14.0 ± 1.4 ^b,1^	22.7 ± 0.4 ^b,2,^*	6.0 ± 0.5 ^b^	11.8 ± 0.9 ^c,1,^*
30	Lab scale	With ice bath	13.5 ± 1.6 ^b,2^	17.3 ± 0.8 ^a,2,^*	3.44 ± 0.12 ^b,2^	6.0 ± 0.5 ^b,3,^*	12.1 ± 1.0 ^b,2^	20.1 ± 1.2 ^b,3,^*	8.1 ± 0.7 ^b,1^	10.6 ± 0.4 ^2,^*
		Without ice bath	18.6 ± 1.3 ^b,1^	23.3 ± 2.0 ^b,1,^*	4.79 ± 0.46 ^a,1^	8.1 ± 0.7 ^b,1,^*	16.5 ± 0.6 ^a,1^	34.2 ± 2.4 ^a,1,^*	6.5 ± 0.5 ^b,2^	13.5 ± 1.2 ^b,1,^*
	Pilot scale		14.2 ± 1.4 ^a,2^	23.9 ± 0.8 ^b,1,^*	3.62 ± 0.24 ^b,2^	7.3 ± 0.6 ^b,2,^*	16.0 ± 0.9 ^a,1^	23.3 ± 2.1 ^b,2,^*	6.6 ± 0.6 ^a,b,2^	13.5 ± 1.1 ^b,1,^*
60	Lab scale	With ice bath	16.2 ± 2.1 ^a,2^	17.1 ± 1.3 ^a,3^	4.11 ± 0.20 ^a,2^	6.8 ± 0.6 ^a,3,^*	17.6 ± 2.0 ^a,1^	22.5 ± 1.3 ^a,3,^*	10.5 ± 0.6 ^a,1^	10.6 ± 0.7 ^3^
		Without ice bath	20.1 ± 0.6 ^a,1^	32.6 ± 2.1 ^a,1,^*	5.20 ± 0.20 ^a,1^	9.5 ± 0.6 ^a,1,^*	14.8 ± 0.6 ^b,2^	26.3 ± 1.5 ^c,2,^*	8.9 ± 0.4 ^a,2^	20.1 ± 1.5 ^a,1,^*
	Pilot scale		15.0 ± 1.4 ^a,2^	26.0 ± 1.5 ^a,2,^*	4.62 ± 0.52 ^a,1,2^	8.3 ± 0.8 ^a,2,^*	14.9 ± 1.5 ^a,b,2^	33.4 ± 2.1 ^a,1,^*	7.3 ± 0.6 ^a,3^	15.1 ± 1.5 ^a,2,^*

For each assay (TPC, TFC, DPPH-RSA and FRAP) different letters for the same UAE technique and vine-cane variety means that extraction time produces statistically significant differences (*p* < 0.05). Different numbers for the same extraction time and vine-cane variety means that different UAE technique originate statistically significant differences (*p* < 0.05). * for the same extraction time and UAE technique means that there are statistically significant differences (*p* < 0.05) between the TN and TR wine-canes varieties.

**Table 2:** Content of the identified phenolic compounds in Touriga Nacional (TN) and Tinta Roriz (TR) extracts obtained by ultrasound assisted extraction (UAE) performed at lab scale without an ice bath after 60 min of extraction; results are expressed as mean ± standard deviation (mg of compound/100 g DW, *n* = 3).

Compound	TN	TR
**Phenolic acids**		
Gallic acid	13.8 ± 0.7	15.1 ± 0.8
Protocatechuic acid	13.7 ± 0.7	10.7 ± 0.5
4-hydroxyphenilacetic acid	4.4 ± 0.2	8.3 ± 0.4
4-hydroxybenzoic acid	6.8 ± 0.3	5.3 ± 0.3
4-hydroxybenzaldehyde	8.6 ± 0.4	9.4 ± 0.5
Chlorogenic acid	11.1 ± 0.6	11.5 ± 0.6
Vanillic acid	4.8 ± 0.2	6.5 ± 0.3
Caffeic acid	6.4 ± 0.3	6.3 ± 0.3
Syringic acid	<LOQ ^a^	<LOQ
*p*-coumaric acid	10.8 ± 0.5	8.9 ± 0.4
Ferulic acid	4.3 ± 0.2	4.4 ± 0.2
Sinapic acid	6.6 ± 0.3	6.6 ± 0.3
Ellagic acid	<LOD ^b^	<LOD
Cinnamic acid	6.5 ± 0.3	7.6 ± 0.4
**∑Phenolic acids**	**97.7 ± 4.9**	**100.4 ± 5.0**
**Flavanols**		
Catechin	35.5 ± 1.8	131.4 ± 6.6
Epicatechin	7.5 ± 0.4	18.1 ± 0.9
**∑Flavanols**	**43.0 ± 2.1**	**149.5 ± 7.5**
**Flavanones**		
Naringin	4.4 ± 0.2	4.9 ± 0.2
Naringenin	2.6 ± 0.1	3.0 ± 0.1
Pinocenbrin	10.3 ± 0.5	10.3 ± 0.5
**∑Flavanones**	**17.3 ± 0.9**	**18.2 ± 0.9**
**Flavonols**		
Rutin	23.3 ± 1.2	21.8 ± 1.1
Quercetin-3-*O*-glucopyranoside	12.8 ± 0.6	14.2 ± 0.7
Myricetin	74.9 ± 3.7	74.3 ± 3.7
Kaempferol-3-*O*-glucoside	9.9 ± 0.5	9.7 ± 0.5
Kaempferol-3-*O*-rutinoside	10.8 ± 0.5	12.4 ± 0.6
Quercetin	20.1 ± 1.0	19.7 ± 1.0
Tiliroside	8.2 ± 0.4	8.0 ± 0.4
Kaempferol	23.9 ± 1.2	22.8 ± 1.1
**∑Flavonols**	**183.8 ± 9.2**	**173.4 ± 8.7**
**Stilbenes**		
Resveratrol	84.0 ± 4.2	153.4 ± 7.7
**∑Stilbenes**	**84.0 ± 4.2**	**153.4 ± 7.7**
**Others**		
Phloridzin	7.9 ± 0.4	8.1 ± 0.4
Phloretin	5.6 ± 0.3	3.9 ± 0.2
**∑Others**	**13.5 ± 0.7**	**12.0 ± 0.6**
**∑All phenolic compounds**	**439**	**617**

^a^ LOQ: limit of quantification; ^b^ LOD: limit of detection.
